# Threat and blame frames in political rhetoric about societal issues lead to neural and political polarization

**DOI:** 10.1038/s41598-026-43389-9

**Published:** 2026-03-20

**Authors:** Elisa van der Plas, Lara Todorova, Karin Heidlmayr, Giedo Jansen, Martin Rosema, Alan G. Sanfey

**Affiliations:** 1 Wellcome Centre for Human Neuroimaging, University College London, London, WC1N 3BG UK; 2 Donders Institute for Brain, Cognition and Behavior, Radboud University Nijmegen, Nijmegen, 6525 EN The Netherlands; 3 Max-Planck Institute for Psycholinguistics, Nijmegen, 6525 XD The Netherlands; 4 Hugo Sinzheimer Instituut, Amsterdam Institute for Advanced Labour Studies, University of Amsterdam, Amsterdam, The Netherlands; 5 Department of Public Administration, University of Twente Enschede, Enschede, 7522 NB The Netherlands

**Keywords:** Societal issues, Neuroscience, Affective polarization, Inter-brain correlations, Normative voting, Neuroscience, Psychology, Psychology

## Abstract

Online content about societal issues like climate change and immigration are often presented via frames of threat and blame. Here, we investigated how exposure to such framing in the context of an online short video-clip impacts voting behavior and associated brain activity. In a large-scale online study of 1825 Dutch participants, we found that online threat and blame framed video-clips increased agreement with the clips themselves but decreased issue voting, that is, voting in line with the intensity of one’s political beliefs. A follow-up fMRI study with 27 participants replicated this behavioral finding. It also showed that video-clips with threat- or blame-frames, compared to neutral video-clips, were represented more dissimilarly across participants in the dorsolateral prefrontal cortex (DLPFC)—a region involved in narrative understanding. These findings suggest that subtle framing of online political content can influence voter decisions and even the fundamental act of communication itself within a society.

**Supplementary Information:**

The online version contains supplementary material available at 10.1038/s41598-026-43389-9.

## Introduction

“Wherever Law Ends, Tyranny begins”

- Locke, Second Treatise of Government.

John Locke’s philosophy defines societal freedom as the natural liberty of an individual to act under no other legislative power than that which is established by consent. If a government violates this trust, it forfeits the legitimacy of its power—and tyranny begins. In this context, Locke’s ideas remain profoundly relevant today.

With the rise in social media and the concomitant fragmentation of information, the traditional influence of central sources of information such as network television or national newspapers has given way to media platforms such as YouTube, TikTok, X and Instagram. On these platforms, modes of manipulation and coercion–often in the form of threat and blame rhetoric–can become so intertwined with fundamental decision processes that the very freedom upon which democracies rest can begin to erode^1–5^. *Threat frames* emphasize societal issues in terms of their danger or negative consequences, often simultaneously bringing them subjectively closer in time or space (e.g., “Climate change is no longer some far-off problem, it is happening here, it is happening now” - Obama^6^). In contrast, *blame frames* focus societal issues by attributing their faults to a third party or outgroup (e.g., “Behind mass immigration, there is terrorism” - Le Pen^7^). Even though such political rhetoric is widespread, it remains poorly understood how such messaging is processed in the brain and subsequently affects political attitudes and behaviour.

While social media platforms provide an important opportunity for a broad constituency across all political ideologies to freely share their theories and concerns about societal issues, often via outspoken and direct conversation^8,9^, the algorithms behind larger social media platforms and generative AI technology may directly promote these frames as they may make content more likely to be clicked on and reshared^10–12^. In addition, work has shown that accusations added to political newsfeeds on X can have a contagious effect on recipients, making recipients significantly more likely to express outrage themselves^13^. This raises the question of whether related exposure to threat and blame framing on social media has lasting neurocognitive effects on people’s capacity to reach consensus, both inter-personally, through (online) social and political discussion, as well as intra-personally, in the form of internal deliberation and normative decision making.

Issue voting —a theoretical model that posits that electoral choice is driven by the intensity of one’s position on key political issues—may be particularly affected by such mechanisms, as it can be strongly influenced by affective responses^14^. Individual differences in emotional susceptibility to political stimuli are theoretically grounded in negativity bias (a tendency for enhanced attention and greater experienced physical arousal to negative versus positive stimuli) which may heighten an individual’s susceptibility to political framing^15–18^. Consequently, negativity bias can transform the computation of issue voting into an emotional response, thereby undermining the model’s assumption that issue positions themselves are the primary drivers of political choice and actions.

The neural architecture of negativity bias is marked by heightened amygdala activation in response to negative stimuli^19^ and by activity in the dorsolateral prefrontal cortex (DLPFC), which correlates with individual^20,21^ and interpersonal^13,22,23^ differences in emotional susceptibility to negatively framed political stimuli. Further work assessing negativity biases also highlights that distinct types of frames may also have distinct effects on issue-voting. For instance, information presented as a threat is experienced with fear, which motivates recipients to gather more information about the threat to evaluate its importance and how to cope with it^24–26^. On the other hand, issues presented with blame frames are experienced with anger, which can lead to stagnated information processing and a focus on quick retaliation against the blamed third party^27,28^, suggesting that in contexts of blame, anger may undermine issue voting pathways.

Here, we extend this prior work by investigating how varied negative framing of political information (specifically, threat and blame) can exploit this negativity bias, and explore the distinct emotional (fear, anger) and associated neural pathways that underlie this effect. We use a causal design to examine whether these effects on neurocognition are due to the exposure itself rather than due to pre-existing tendencies. This remains a challenging question to answer experimentally, for two primary reasons. Firstly, because it requires the introduction of naturalistic social media content in experimental functional Magnetic Resonance Imaging (fMRI) settings, where the timing of stimuli needs to be precisely manipulated in order to extract relevant Blood-Oxygen-Level-Dependent (BOLD) signals; and secondly, because more polarized individuals are more likely to watch partisan news^29^, which makes it difficult to parse tendencies that may be pre-existing versus those that are caused by the content itself.

To address these challenges, we generated 36 short naturalistic video-clips about three contemporary salient societal issues (climate change, immigration, and healthcare) and showed one of these video-clips to each of 1825 Dutch *citizen*s who participated in an online survey. We experimentally counterbalanced the wording of the video-clip to either present the societal issue in a neutral context (e.g., “Sometimes immigrants get financial support to survive”) or with language that characterises how such issues are often discussed in public and political debate (e.g., “Immigrants take advantage of our tax money”). The treatment consisted of two further categories: video-clips with a threat frame emphasized the negative consequences of the issue (e.g. “Immigration will be detrimental to your career chances”); video-clips with a blame frame focused on its cause (e.g. “Companies are taking advantage of the sick, and this must stop now”). Importantly, the timing of words, the narrator’s voice, and the specific images used were as much as possible equivalent across all experimental conditions. Overall, all three types of video-clips had the same broad visual and auditory properties (Supplementary Materials 1.1). Across two studies conducted in 2017, we tested the effects of this counterbalancing in a between- and within-subject design, respectively.

In Study 1, participants rated their attitudes to the seven main political parties that existed in The Netherlands at the time of recruitment, both before and after within-subject video-clip exposure. The Dutch political system provides a critical test bed for our research questions due to its multi-party system, which is characterized by “issue ownership” of some parties over specific issues. First, issue voting theory would predict that affect elicited by an issue will shape attitudes towards its corresponding “owning” party. In particular, the right-wing Party for Freedom (Partij voor de Vrijheid or PVV) is often perceived to own the issue of immigration, while, at the time of the survey, the Green Party (known as GroenLinks, or GreenLeft) is often perceived to own the issue of climate change. Second, The Netherlands has seen a stark rise in populist rhetoric and political polarization over the last years^1,30^. This offers a context that allows to test any moderating effect of such rhetoric—specifically, whether threat and blame frames disrupt affective pathways of issue voting, thereby weakening the link between an individual’s issue positions and their political attitudes and actions.

In Study 2, we used these video-clips in a within-subject fMRI design on 27 participants to examine the neural correlates of political framing effects. Participants were exposed to a set of video-clips while undergoing fMRI. We used inter-subject correlation (ISC) analysis to quantify neural synchronization, a measure of how consistently specific neural regions are activated across participants when viewing the same particular content. Based on prior research, we focused primarily on the DLPFC, an area involved in interpersonal perspective-taking, narrative comprehension, and political reasoning^22,23^^,31,32^. We hypothesized that threat and blame framing would alter neural synchronization patterns in the DLPFC, potentially disrupting shared neural representations and increasing partisan processing of political information.

## Results

To summarize our results, in Study 1 we found that presenting a video-clip about societal issues using threat- or blame-frames evoked greater negative arousal than when it was presented in neutral wording. While negative arousal is positively associated with video-clip sharing and issue importance, across both studies we find that blame frames dissociate issue importance from sharing behavior. Our neuroimaging results further demonstrated that video-clips were processed more dissimilarly among distinct participants when they were presented using threat- or blame-framing than when the same information was presented in a neutral context. This effect was most pronounced for video-clips with blame framing, which led to greater desynchronization of neural representations among voters with pre-existing differences in political ideologies. Taken together, these results suggest that blame framing in particular may lead to neural and behavioral correlates of political polarization.

### Study 1

**Fig. 1:**
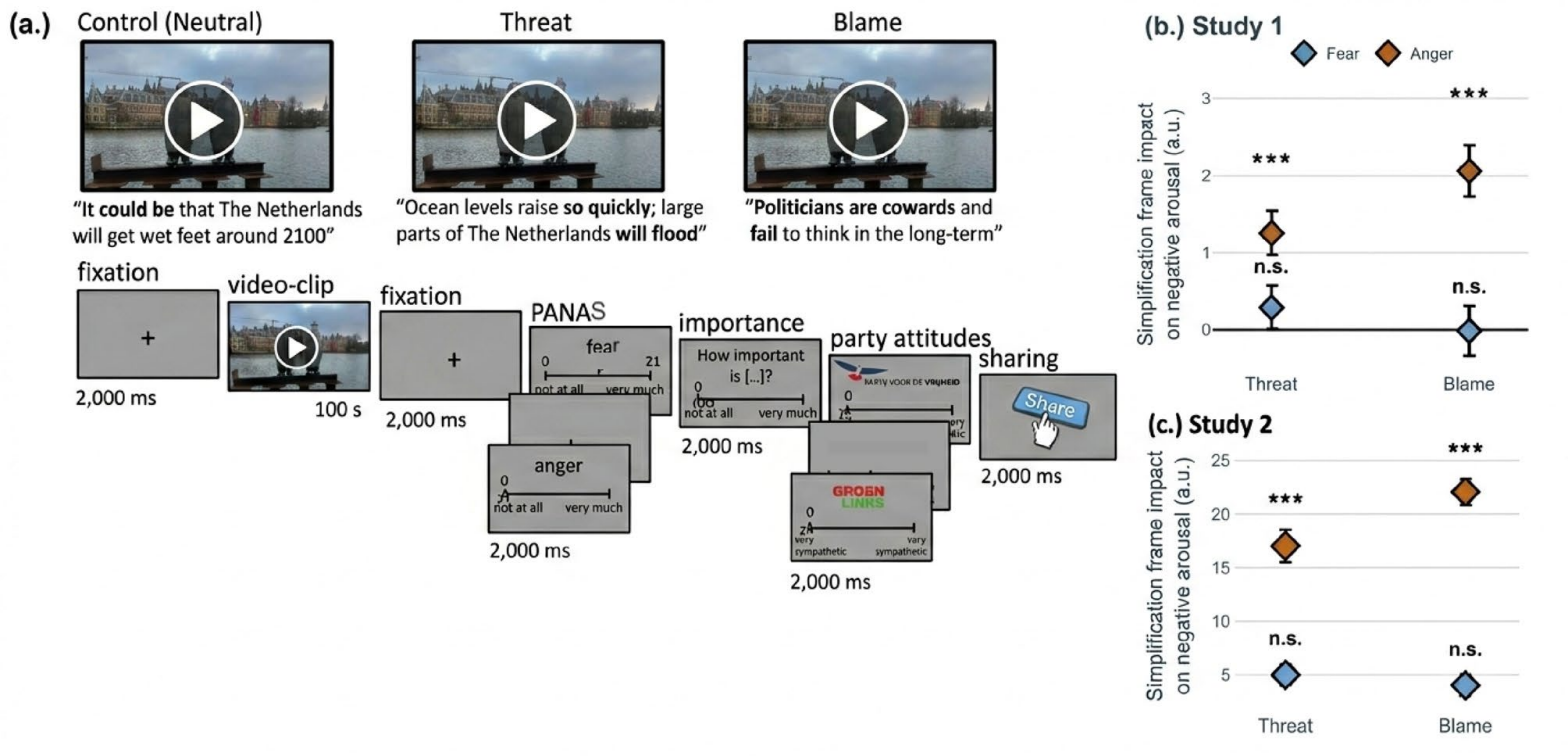
Experimental paradigm and manipulation-check. (**a**) Participants were instructed to watch one of nine (Study 1) or all 36 (Study 2) short video-clips about a societal issue which text was presented across three conditions (threat, blame or neutral) and (1) rate their emotions on a twelve-item emotions scale; (2) indicate the importance of the societal issue; (3) rate how much they agreed with the statements in the video-clip (Study 1) or rate how much they wanted to share the video-clip online (Study 2); and (4) rate their attitudes towards the corresponding political party. All responses had a 2.000 ms time limit. (**b**) Standardized beta coefficients for the impact of threat and blame frames (vs. neutral) on self-reported emotions (fear in blue, anger in red) in comparison with the neutral condition in Study 1 and Study 2 (**c**). n.s. *P* > .05, *P < .01, **P < .001, ***P < .001.

In Study 1 we analyzed the data of 1825 Dutch adults (53.37% female, mean age = 54.43, Standard Error from the Mean (SEM) = 0.50), whose demographics indicate reasonable representativeness with the Dutch population with regard to age, gender, education, employment status and political attitudes. Participants were randomly contacted from a nationally representative respondent pool. At the start of the experiment, we assessed participants’ attitudes towards the seven main political parties in The Netherlands (at the time of recruitment). Then, each participant was shown one short (100 ± 5 s) video-clip, where the topic (immigration, climate change, or health care) and frame (neutral, threat, or blame) were counterbalanced between participants (Fig. 1a; Supplementary Materials 1.1). All models included standard demographic and political-psychological control variables—covariates known to predict political attitudes based on prior electoral research (e.g. Dutch National Election Studies^33^), such as personality traits (Big Five), education level, gender, political affiliation, pre-test stances on societal issues, authoritarianism, political skepticism, social media use for political news and self-reported interest in politics (see Supplementary Materials 1.2 for a full description of the demographics). Immediately after watching the video-clip, participants reported their subjective emotional state using the Positive Affect Negative Affect Scale (PANAS;^24^). From this scale, we derived measures of two negative emotions (fear, anger) alongside aggregate scores for overall negative and positive affect (see Supplementary Materials 1.3). In Supplementary Table 1.2 and 1.3, we provide the statistical information and ranges for primary measures and the full regression models, respectively.

Video-clips presented with a simplification frame (i.e. threat or blame) evoked more negative affect than video-clips presented with a neutral frame (main effect of frame: βthreat = 0.41, *SE* = 0.127, *p* = .001, 95% CI [0.163, 0.660]; βblame = 0.57, *SE* = 0.148, *p* < .001, 95% CI [0.283, 0.863]). Anger was significantly higher in both the threat condition (βthreat = 1.26, *SE* = 0.288, *p* < .001, 95% CI [0.694, 1.823]) and the blame condition (βblame = 2.06, *SE* = 0.335, *p* < .001, 95% CI [1.403, 2.717]) compared to the neutral condition (Fig. 1b; Supplementary Materials 1.4). In contrast, fear was not significantly affected by the threat (β = 0.29, *p* = .303) or blame (β = -0.01, *p* = .975) frames.

We next investigated whether the negative arousal elicited by political video-clips predicts attitudes toward specific political parties. A linear regression model predicting attitudes towards the right-wing, anti-immigration, party (PVV) revealed that negative affect was a significant positive predictor (β = 0.34, *SE* = 0.084, *p* < .001). This relationship was moderated by topic, with the effect being significantly weaker for environmental (β = – 0.28, *SE* = 0.129, *p* = .029) and healthcare (β = – 0.38, *SE* = 0.120, *p* = .001) topics as compared to immigration topics. A marginally significant three-way interaction (*p* = .058) was observed, suggesting a potential trend where the combined effect of immigration importance and negative arousal on PVV attitudes was most pronounced in the blame condition.

Similarly, party attitudes towards the left-wing, green, party (GL) could be predicted from pre-existing climate change issue importance (main effect of climate issue importance: β = 0.69, *SE* = 0.121, *p* < .001). A marginal interaction with negative affect indicates little evidence for an interaction with the video-clip topic on GL attitudes (*p* = .075), while the three-way interaction here indicates that the blame frame undermines the relationship between climate importance, negative affect and GL support (climate importance × blame frame × negative affect: β = – 0.10, *SE* = 0.035, *p* = .005; Supplementary Materials 1.5).

**Fig. 2:**
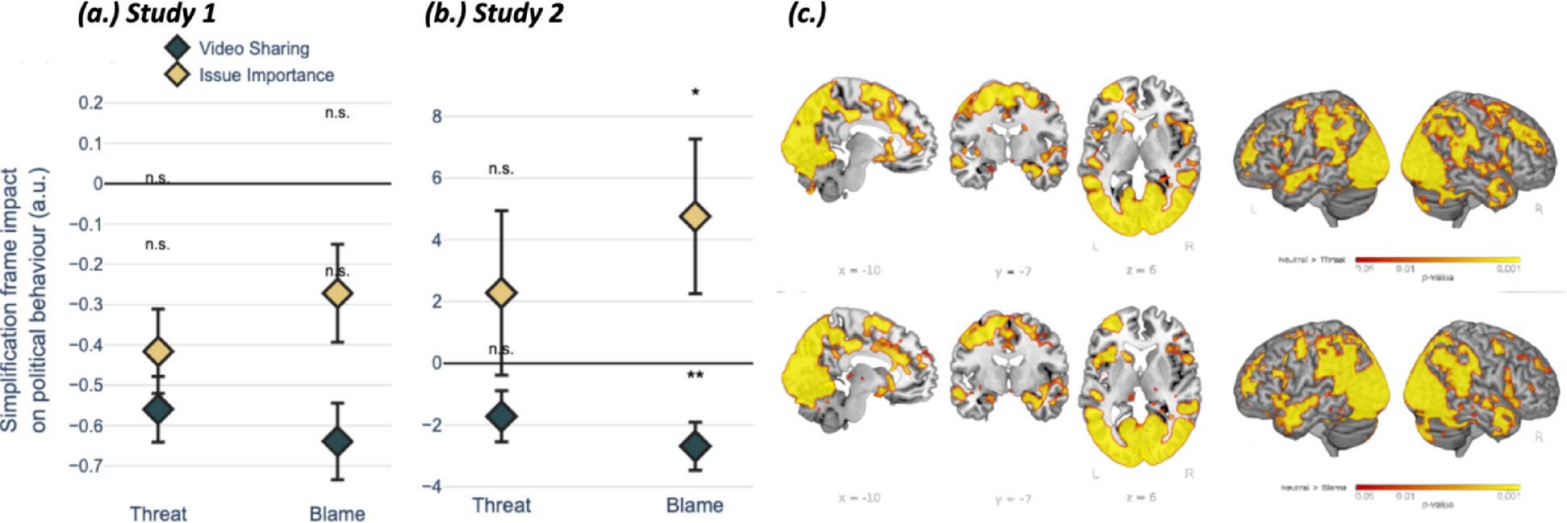
Simplification frames increase arousal but suppress sharing behavior. (**a**) Study 1: Standardized beta coefficients (adjusted by -0.5 for visualization) show the impact of threat and blame frames (vs. neutral) on political behavior. Blame frames significantly reduced video sharing (dark blue) but did not significantly affect issue importance (yellow). (**b**) Study 2: Replicating Study 1 in a within-subjects design, both threat and blame frames significantly reduced actual sharing behavior (blue), with no significant direct effect on issue importance (yellow). All y-values are adjusted by – 0.5 for visualisation. (**c**) Effects of threat and blame frames on inter-subject correlations. Clusters with significantly higher inter-subject correlations between individual participants’ hemodynamic time courses during neutral video-clips than when the video-clip was framed with either threat or (**d**) blame. Error bars represent the group means ± 1 Standard Error. Significance markers represent the contrast between the threat/blame and neutral frame conditions from a linear regression model. n.s. *P* > .05, *P < .01, **P < .001.

Regarding political behavior, negative affect was a strong positive predictor of both video-clip sharing (β = 0.10, *SE* = 0.013, *p* < .001) and perceived issue importance (β = 0.02, *SE* = 0.010, *p* = .011). However, blame framing directly reduced sharing intentions (β = – 0.20, *SE* = 0.084, *p* = .016) without a significant direct effect on issue importance (*p* = .350). Threat framing did not have a significant direct effect on either sharing (*p* = .167) or importance (*p* = .079; Fig. 2a). Taken together, these results suggest different psychological pathways for political engagement across distinct framing contexts. To explore this further in a within-subject design, we conducted Study 2.

### Study 2

In Study 2 we analyzed the data of 27 Dutch adults (50.84% female). Building on the evidence from Study 1, we utilized broadly the same paradigm as in Study 1, but here presented the video-clips using a within-subject design. That is, every participant saw 36 video-clips (12 unique stimuli x 3 frame conditions), rating their preferences and behavior after each video-clip. To enhance ecological validity, here we operationalized video-clip sharing as the amount of “applause” (a continuous reward measure) participants awarded to each clip, which quantifies a behavioural proxy for willingness to support a stimulus^34^. All models controlled for education level, gender, age, political affiliation, pre-test stances on societal issues, authoritarianism, and political skepticism (see Supplementary Materials 1.2).

#### Behavioural findings

Replicating and extending Study 1, threat and blame video-clips elicited stronger negative arousal than the neutral frame (main effect of framing: βthreat = 10.96, SE = 2.123, *p* < .001, 95% CI [6.794, 15.126]; βblame = 13.09, SE = 1.999, *p* < .001, 95% CI [9.167, 17.015]). Anger responses were significantly higher in both the threat (βthreat = 13.70, SE = 2.377, *p* < .001, 95% CI [9.036, 18.364]) and blame (βblame = 21.24, SE = 2.238, *p* < .001, 95% CI [16.844, 25.631]) conditions. In contrast to Study 1, fear responses also significantly increased under both threat (βthreat = 8.22, SE = 2.543, *p* = .001, 95% CI [3.230, 13.210]) and blame framing (βblame = 4.94, SE = 2.395, *p* = .039, 95% CI [0.244, 9.644]; Fig. 1c; Supplementary Materials 2.1; and Supplementary Table 2.1 and 2.2 for the statistics and ranges of the primary measures and the full regression models, respectively).

Next, we tested the impact of framing on issue importance and willingness to share the video-clip with others. Replicating Study 1, negative affect had a strong positive association with both video-clip sharing (βsharing = 0.08, SE = 0.013, *p* < .001) and issue importance (βissue importance = 0.44, SE = 0.039, *p* < .001). Crucially, and again replicating Study 1, blame framing significantly reduced sharing (βblame = – 3.25, SE = 0.780, *p* < .001) without a significant effect on issue importance (*p* = .853). Threat framing also significantly reduced sharing (β = -2.11, SE = 0.821, *p* = .010) with no significant effect on importance (*p* = .429; Fig. 2b). Together, these results robustly replicate the behavioral findings of Study 1: while simplification frames, especially blame, are potent at eliciting negative arousal (particularly anger), they paradoxically directly suppress the very sharing behavior that this arousal otherwise promotes.

#### Neural findings

An additional goal of Study 2 was to examine the change in how issue rhetoric is neurally processed as a function of simplification framing (i.e. threat and blame). To assess this question, we conducted inter-subject correlations (ISC) on functional brain imaging data. This analysis revealed a reduction in ISC for video-clips on societal issues that were presented with a simplification frame, as compared to those presented with a neutral context. This reduction in ISC suggests that there was more *dissimilar* neural processing across participants in the framing conditions. In particular, activation in the dorsolateral prefrontal cortex (DLPFC) was more dissimilar across participants when the societal issue was presented with the two simplification frames as compared to the same video-clip presented neutrally (Supplementary Materials 2.2; Supplementary Table 2.3; Fig. 2c). ISC was reduced bilaterally when comparing neutral with threat video-clips, and left-dominantly when comparing neutral with blame video-clips in the bilateral inferior frontal gyrus, bilateral medial and lateral parietal cortex, bilateral occipital cortex, bilateral anterior and middle temporal cortex, bilateral thalamus, amygdala, putamen and caudate (Supplementary Table 2.4).

**Fig. 3:**
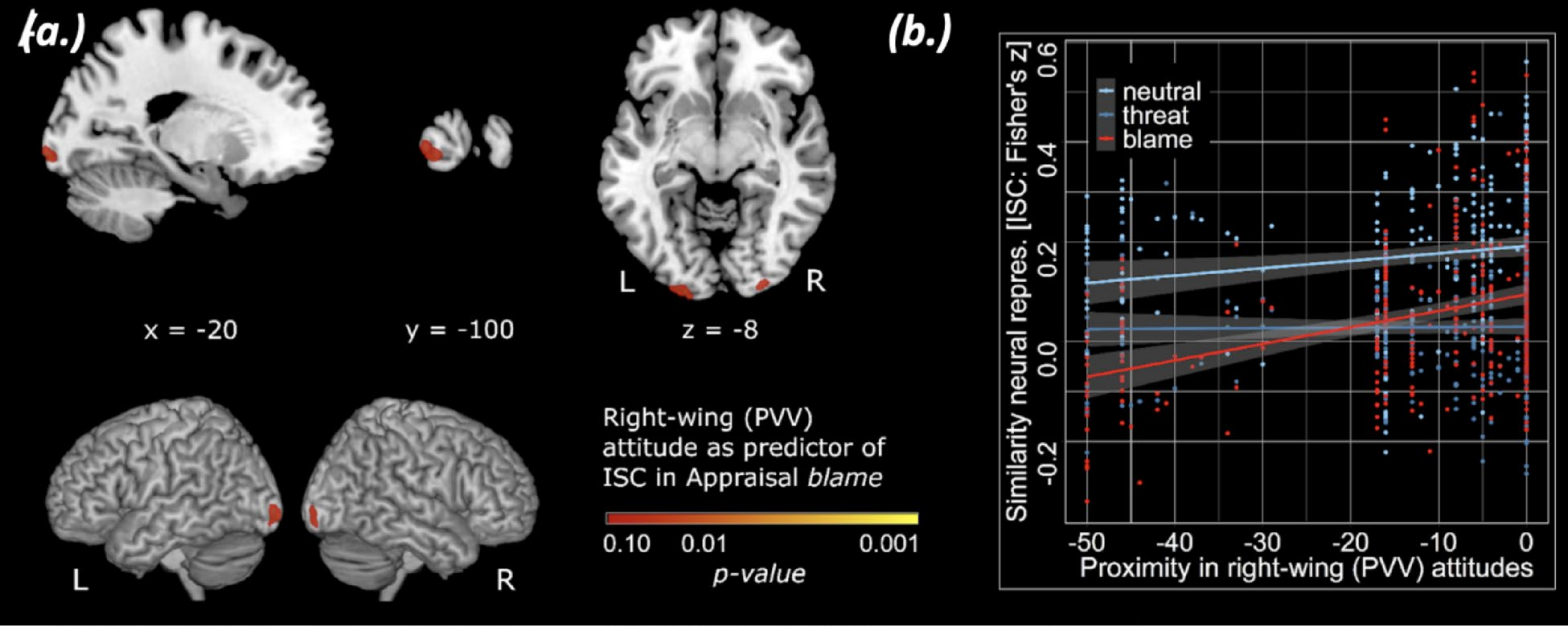
Blame exposure leads to political polarization. (**a**) The proximity (negative numerical distance) between individual scores on right-wing party attitudes as a predictor of the ISC in frontal and occipital regions for threat and blame video-clips in comparison to neutral video-clips. (**b**) Effects of framing conditions on inter-subject correlations across individual scores on right-wing party attitudes. Error bars represent ± SEM from the mean. Whole-brain significance (P-value) map, FWE-corrected within and across contrasts.

Next, we conducted multiple regressions on inter-brain synchrony with political party attitude as predictors. We identified several relevant predictors of political party preferences on ISC for video-clips that were presented with a simplification frame. Firstly, the proximity (negative numerical distance) between individual scores on right-wing PVV attitudes was found to be a predictor of the frontal and occipital ISC for video-clips with blame framing, as compared with threat and neutral contexts (Fig. 3a, Supplementary Materials 2.3). A similar pattern was observed for ISC analyses on Green Party attitudes for video-clips with the topic of climate change (Supplementary Fig. 2.1; Supplementary Table 2.5). In line with the behavioral finding of Study 1, blame framing in particular exacerbated neural desynchronization among participants that had more dissimilar right-wing party (PVV) attitudes before video-clip exposure (Fig. 3b).

Together, these findings suggest that societal issues are more dissimilarly represented across participants in both frontal and occipital regions when they are presented with threat- and blame-frames. In addition, societal issues were most likely to cause political polarization among participants with more positive attitudes towards the right-wing party (PVV) when the corresponding societal issue was framed with blame rather than threat. This suggests that exposure to blame framing might undermine a shared understanding of societal issues, especially when these deviate from one’s pre-existing political attitudes.

## Discussion

In this set of studies, voters viewed short political video-clips about three important societal issues, climate change, immigration, and health care. Though visually identical, the issues were presented to participants using narration invoking different forms of rhetoric, either presenting the issue neutrally or adopting one of two simplification frames, namely blame and threat. Our primary question was whether presenting the same societal issue via these negative frames would cause shifts in information processing pathways and lead to increased political polarization.

In Study 1 on 1825 voting Dutch voters, we indeed found that video-clips about societal issues evoked stronger arousal and issue importance when presented using threat and blame framing than when presented neutrally. However, distinct simplification frames may have dissimilar effects on behavior. While negative arousal strongly predicted both issue importance and sharing behavior, threat and blame framing actually suppressed video-clip sharing, without affecting self-reported issue importance. Given the exploratory nature of this higher-order interaction and its marginal significance in the large-scale dataset of Study 1, this finding should be interpreted with caution and requires replication. This dissociation was particularly pronounced for blame framing—despite eliciting the strongest anger responses it consistently inhibited public sharing behavior. The within-subject design of Study 2 also allowed us to explore the neural information processing pathways involved. Behaviorally, we replicated the finding that blame led to participants being less willing to share and discuss the video-clip with others, without affecting corresponding issue importance. Additionally, the DLPFC—an area involved in narrative understanding^35–37^—more dissimilarly represented the same content across participants when the issue was presented with threat- and blame-frames. Intriguingly, this effect was moderated by blame about issues that participants already had strong political attitudes towards, on both the right and left ends of the political spectrum, which had the effect of driving those with already dissimilar political views even further apart. Together, these findings suggest that framing complex societal issues in terms of threat or blame may hinder shared processing and understanding of its corresponding importance and solutions.

The emotion-laden rhetoric examined in this study is commonly encountered in politics, both in legislative bodies and in social media, and is often used to engage the broader public^38,4,5^. While correlational research has documented a discrepancy between societal issue frames that elicit political arousal and those that affect political action-taking^39–43,25^, the causal impact of subtle rhetorical variations on political attitudes and their neural processing remains unestablished. Our findings reveal profound differential effects: the blame frame demonstrates a strong polarising effect on political attitudes by consistently suppressing the willingness to share political content. This is in line with work that shows that anger, and not fear, is a key driver of attitude shifts towards more dominant political figures^44^.

Our supplementary univariate neural analyses provide additional insight into the distinct cognitive patterns that each of the frames elicited (Supplementary Materials 2.2). Distinct frames activated different neural regions, including the precuneus, occipital cortex and (para)cingulate gyrus—regions associated with processing emotions, behavioral regulation, self-awareness and episodic memory^45–47^. This suggests that emotion-laden political communication may hinder core self-regulatory processes that facilitate shared understanding^48^ and social perspective-taking^49^. Our ISC analyses are in line with this interpretation. By leveraging a previously validated neural characterization of political polarization^22,23^, we showed that emotion-laden rhetorics selectively decrease a shared understanding of political context in the dorsolateral prefrontal cortex (DLPFC). The DLPFC is involved in narrative understanding^35–37^, descriptive understanding of others’ social behaviors^32^ and social influence^31,50^. Our fMRI results suggest that blame-induced anger may specifically disrupt integrative processes–indicating that blame frames in political rhetoric may affect self-regulatory processes involved in shared understanding. These results build upon prior studies that show that political partisans process emotionally laden political information more dissimilarly in the DLPFC^51,52,22^. We show here that emotional information can have causal polarizing effects within voters across a range of “typical” left-wing (e.g., climate change) and right-wing (e.g., mass immigration) societal issues (see Supplementary Materials 1.5 and Supplementary Materials 2.3 for the behavioral and neural effects on Green Party attitudes in Study 1 and 2, respectively). In addition, we corrected for baseline political attitudes, personality traits, demographics, and agreement with the main opinion expressed in the video-clip. We extended this finding by showing that the same voter processes the same information more dissimilarly from other voters when the information was framed with threat or blame than when it was presented neutrally, indicating that these effects hold across partisans and non-partisans alike. Our findings lend support for political science research indicating that the narrative frame in which political information is presented, rather than information about societal issues itself, plays a role in “spreading” political attitudes and polarizing voters^44,39,41,53,43,25^). Critically, we demonstrate this is not merely a correlation: exposure to blame frames can directly alter neurocognitive processing and shift political attitudes. In a media landscape where engagement-driven algorithms may preferentially amplify blame-laden content^13^ this may not only intensify polarization but also systematically favor support for more aggressive policies and authoritarian leadership styles^54^.

A potential alternative explanation is that blame frame may not have elicited anger towards the political culprits depicted in the videos, but rather provoked frustration with the video-clips themselves. This could account for the distinct emotional pathways observed between issue importance and negative affect in predicting party attitudes in Study 1. However, some additional analyses nuance this interpretation. The blame condition evoked a behavioral response that resembled prior attitudinal work on individuals that score high on self-reported authoritarianism—a tendency to grant political figures full responsibility over societal issues^55^. High authoritarian individuals are more likely to report stronger feelings of anger towards political representatives and societal issues^40,56,25,25^ and are less likely to consider other viewpoints in their formation of attitudes about the issue^57,51,58^ than those who score lower on political authoritarianism^59–61^. Political authoritarianism is further associated with reduced openness to dissimilar political opinions^62,63^ and a reduced tendency to openly challenge one’s own political attitudes online^64,65^—behavioral features that resemble those that we found in the blame condition (see behavioral analyses on authoritarianism scores in Supplementary Materials 2.4). This interpretation was further supported by our fMRI findings, where trait authoritarianism was correlated with reduced ISC during especially the blame condition (see neural analyses on authoritarianism in Supplementary Materials 2.4 and Fig. S2.2). This suggests that authoritarian appraisals may work directly on political polarization, adding an additional contagious effect of being on the receiving end of blame rhetoric on social media from human or genAI sources.

In conclusion, we demonstrate evidence that rhetorical use of threat and blame framing approaches towards societal issues may have causal effects on the way recipients form their political attitudes. Emphasizing the consequence (threat) or cause (blame) of societal issues can make the issue more negatively arousing, and therefore more salient, but may also predispose people to neural and behavioral characteristics of political polarization. In particular, blame has strong causal effects on recipients’ ability to reach a shared understanding of the causes and consequences of societal issues—something that is deemed vital to well functioning democracies and may be, inadvertently or otherwise, promoted in modern algorithms underlying text generation or social media recommendation systems. These results highlight the societal risk for excessive use of emotion-laden political communication, as these may color reality in ways that are superficially appealing but may also directly hinder reaching consensus about issues that, eventually, can only be resolved through collaboration and open discussion.

## Materials and methods

### Study 1

#### Participants

This study was approved by the ethics committee of the University of Twente (ethical approval number: UT0106), and conducted in accordance with the Declaration of Helsinki. All methods were performed in accordance with relevant guidelines and regulations, and all participants provided informed consent.

We contacted 3,959 participants from a nationally representative respondent pool from I&O, a nationwide professional survey organization which maintains a large, nationally representative respondent pool of over 25,000 Dutch adults. The company randomly contacted 3.959 participants from their respondent pool with a short description of the study and the lottery incentives that were raffled among participants. Participants were told that they could take part in a “survey regarding a number of societal issues” that was restricted to laptop and tablet users. Nonrespondents were sent a reminder after one week and the survey was closed after thirteen days (response rate = 47%). Inclusion criteria were Dutch nationality and having good-to-corrected hearing and eyesight. Directly after the manipulation, we included a “catch-trial” that asked what the main topic of the video-clip was. Respondents that reported not to have been able to both hear and read the text (1%; *N* = 24) and those that were unable to indicate what the main topic of the video was on the catch trial (2%; *N* = 32) were excluded from further analyses, towards a final dataset of 1825.

Demographics indicate reasonable representativeness to the Dutch population with regard to age (35% in the 35–49 age range), gender (53% female), education (37% attended middle-level applied education), occupancy (unemployment rate is 5%). Due to the inclusion criterion, Dutch nationality was overrepresented (100% compared to 80% in the general population). Reported votes on the Dutch elections in March 2017 closely matched national results. The left-wing Green Party/GL was slightly overrepresented (11%, compared with 9% in the general population) and the right-wing Party for Freedom/PVV was slightly underrepresented (7%, compared with 13% in the general population).

#### Paradigm

After having given written informed consent, we assessed participants’ political ideology (on a 7-point Likert scale from “left” to “right” and a separate “I do not know’’ option) and attitudes towards the seven main Dutch political parties at the time of recruitment (on a 21-point Likert ranging from “very unsympathetic” to “very sympathetic” and for each party a separate “I do not know” option). Participants then completed a Dutch translation of the political skepticism scale^66^, their agreement with three issue positions maintained in each of the video-clips topics (e.g., “We should strive for less immigration in The Netherlands” for immigration video-clips; “The Dutch government should do more to prevent climate change” for climate change video-clips; “The Dutch health care system is poorly organized” for health care video-clips), a Dutch translation of the authoritarianism scale (Scheepers et al. 1990) and a Dutch translation of the 10-item BIG5 personality inventory (Denissen et al., 2008). Similar to other studies examining political information processing (e.g.,^4,67^), covariates were selected for their well-established theoretical relevance to political attitudes and information processing. Specifically, we controlled for demographics (age, gender, education), stable psychological traits (Big Five personality, authoritarianism), and political predispositions (ideology, pre-test party attitudes, skepticism, media use). These variables are standard predictors in models of political behavior (e.g., Dutch National Election Studies^33^). After this, participants were counterbalanced across 3 × 3 between-subject conditions that determined the *topic* of the video-clip (i.e., 33% of participants watched a video-clip about immigration, 33% about climate change and 33% about health care) and the *simplification* frame (i.e. 50% of participants watched their video-clip with a *threat* frame, 25% with a *blame* frame, and 25% with a neutral frame). Participants were then randomly assigned to one of nine conditions resulting from the 3 (topic: immigration, climate change, healthcare) x 3 (frame: threat, blame, neutral) between-subjects design. The assignment was counterbalanced to ensure equal sample sizes across the nine cells, which is a form of constrained randomization that improves design efficiency for testing interactions. The unequal distribution of frame conditions (50% threat, 25% blame, 25% neutral) was implemented to increase power for detecting effects of the threat frame, which was hypothesized to be more prevalent in common discourse, while still allowing robust comparisons of blame and neutral frames against the larger threat baseline.

Finally, after exposure to their assigned video-clip, participants evaluated related political statements using a 21-point response scale (-10 to + 10). This scale was selected to maximize variance and sensitivity in detecting subtle attitudinal shifts^68^, while its symmetrical design provided clear contrasts for evaluating opposing statements.

After having seen the video-clip, participants rated what emotions they experienced while watching on a Dutch translation of the twelve-item Positive and Negative Affect Scale (PANAS; on which six positive and six negative emotions were rated on a 21-point Likert from “not at all” to “very much”; Crawford & Henry, 2004); how important they considered the societal issues of climate change, immigration and health care (on a 21-Likert scale from “very unimportant” to “very important” and a separate “I do not know” option, which responses were re-coded such that higher scores represent higher importance) and their attitudes towards the seven main political parties in The Netherlands at the time of recruitment.

#### Video-clips

Nine video-clips were created for the purpose of this experiment using Adobe Premiere Pro (version 13.1.2, https://www.adobe.com/products/premiere.html) following a strict template of ten sentences of each ten words, which were each followed by a jittered inter-sentence interval of 2–4 s. The imagery of the video-clips was kept equal across conditions, the only difference was the specific wording used to emphasize different emphases which was sourced from publicly available content and presented as subtitles and with a professional voice-over to account for illiteracy (see *Supplementary Materials 1.1* for a full description of the video-clips, imagery and for the traditional and English translated text per video-clip; the video-clips themselves are available upon request). The narration did not directly refer to the manipulated emotions or frames.

#### Statistics

We computed main composite scores of questionnaire items and computed a factor analysis on the emotion ratings in SPSS (version 25.0.0.1; https://www.ibm.com/products/spss-statistics; see *Supplementary Materials 1.3*). Then we exported the dataset as a text file for behavioral analyses in R (version 1.1.463, https://www.r-project.org/)*.* To test the effect of the condition [1: neutral, 2: *threat*, 3: *blame*] and video-clip topic [1: immigration, 2: health care, 3: climate change] on standardized composite negative affect, fear and anger scores while controlling for the covariates (age, gender [-0.5: male, 0.5: female], education 4:, standardized left-right political ideology, standardized attitudes towards the Green Party (GL) and Party for Freedom (PVV) before the experiment, standardized video-clip agreement and standardized composite political authoritarianism scores. These tests were conducted with linear regression models using the ‘lm’ function in R (version 3.3.3) and we plotted the behavioral data and the output of the model fits using the GGPlot2 package. All models include a random effect at the participant level and all statistics are computed at the group level. We obtain *P*-values by fitting ANOVAs to the regression coefficients. We report degrees of freedom (*df*), *P*-values, estimated beta-coefficients (β) and their standard error of the mean (SEM) of the associated contrasts.

### Study 2

#### Participants

This study was approved by the ethics board of Radboud University Medical Centre (ethical approval number: 2014 − 288), and conducted in accordance with the Declaration of Helsinki. All methods were performed in accordance with relevant guidelines and regulations, and all participants provided informed consent.

*N* = 31 Dutch adults were recruited from a university online participant recruitment platform and gave informed consent before experiment onset. Inclusion criteria were Dutch nationality, proficient Dutch reading skills, and normal or corrected-to-normal vision and hearing, and being eligible to vote at the Dutch general elections. Exclusion criteria were giving the same answer on all trials (thus suggesting non-engagement with the material) and completing at least 75% of the experiment. These criteria led to the exclusion of *N* = 4 participants for a total of *N* = 27 participants (of which 50.84% female, M_age_ = 23.56, SEM = 1.38).

#### Paradigm

Participants watched all video-clips in counterbalanced order using the Psychtoolbox software (Brainard, 1997) in MATLAB (MathWorks, version 2019b, http://psychtoolbox.org/) in MATLAB (MathWorks, version 2019b; https://nl.mathworks.com/products/matlab.html). After each video-clip, participants were asked: (1) How likely they were to share the video-clip on YouTube; (2) How important they thought the societal issue in the video-clip was (on a scale from 0 “Not at all” to 100 “Very much”); and (3) to report their negative arousal, using a Positive and Negative Affect Scheme (on a scale from 0 “Not at all” to 100 “Very much”; Crawford & Henry, 2004). All questions were answered using a scanner-compatible button box with a response time-limit of 2,000 milliseconds (ms.). Responses to share the video-clip online were made by repeatedly pressing a button in a time window of 2,000 ms. Participants were instructed that the video-clip that would receive the greatest number of shares (button presses) would be posted on YouTube.com. There was no deception involved in this study, except for that the most popular video-clip was removed from YouTube.com several months after completion of this study.

#### Behavioral statistics

See Study 1.

#### MR image acquisition

Participants were invited to lie in a 3T Skyra MRI system (Siemens Magnetom) where a Finger Tapping Task was conducted (Witt et al., 2008) to localize the motor cortex. After this, we collected an anatomical scan and then acquired functional T2* weighted echo-planar images (multi-band, gradient-echo, repetition-time TR = 1500 ms, echo-time TE1 = 12.40ms, TE2 = 34.30 ms, TE3 = 56.20 ms 0.66 ms echo spacing, 1804 hz/Px bandwidth, generalized auto-calibrating partially parallel acquisition (GRAPPA), acceleration factor 3, 32 channel brain receiver coil). In total, 51 axial slices were acquired (2.5 mm thickness, 2.5*2.5 mm in plane resolution, 210 mm field of view (FOV) whole brain, anterior-to-posterior phase-encoding direction). Field map images were acquired using 3T Skyra MRI system (Siemens Magnetom), T2* weighted echo-planar images (gradient-echo, repetition-time TR = 576ms, echo-time TE1 = 4,30ms, TE2 = 6,76ms, 804 hz/Px bandwidth, 32 channel brain receiver coil).

#### Preprocessing of functional MR imaging data

Preprocessing was performed using SPM12 (Wellcome Trust Center for Neuroimaging, University College London, UK). First, we combined echoes by weighting their TE*tSNR contributions, which were further averaged. In order to correct for heterogeneities of magnetic field, we used field map (phase and magnitude) to calculate the voxel displacement map (vdm) for each condition. In addition, we used estimated vdms to correct functional scans while they were realigned to the first scan of the first run, with further realignment to the mean scan. Realigned functional scans were spatially normalized to the Montreal Neurological Institute (MNI) space (Collins et al., 1994; Evans et al., 1994) without changing the voxel size. Normalized data were smoothed spatially with a Gaussian kernel of 6 mm full width at half-maximum. The anatomical scan was co-registered to the mean functional scan estimated after realignment. We further segmented co-registered anatomical scans to extract the whole brain gray matter mask.

#### Modelling of functional MR imaging data

Inter-subject correlation (ISC) analyses^69^ were conducted to assess the similarity of stimulus- and task-related time courses of brain activity across individuals. For stimuli with an extended time course and a complex BOLD response, an inter-subject correlation (ISC) approach can give valuable insight beyond conventional GLM analyses (*Supplementary Materials 2.2*). That is, given that the similarity of the hemodynamic response time courses across participants is assessed in this approach, a model of the BOLD response at a particular time point during an extended stimulus is not required. Brain areas that show high ISC values between participants are considered to be involved in processing shared stimulus- or task-specific information across these participants (Hasson et al.^69^; for reviews, see^70,71^).

For the ISC analysis, the functional data were preprocessed similarly as the GLM analyses. Before calculating ISC maps, six standard motion parameters were regressed out from the preprocessed data. The preprocessed and cleaned functional data in MNI space were then split into stimulus-specific segments, excluding the first 8 volumes (12 s) to deal with the initial hemodynamic transient signal^72,73^. The segmented functional data were masked with a standard MNI brain mask to exclude non-brain voxels. Next, voxel-wise Pearson correlation maps for all participant pairs and stimuli were calculated and normalized (Fisher’s *r*-to-*z* transform) using in-house customized code in Matlab. All normalized pairwise correlation maps were then averaged per framing and topic condition and subjected to statistical analysis.

In order to account for spatio-temporal dependencies in the data, the averaged ISC maps were analyzed with a (Wilks’ lambda) permutation test (1,000 permutations) with tail approximation (*P* < .10), using FSL’s PALM (Permutation Analysis of Linear Models; Winkler et al.^74^, https://fsl.fmrib.ox.ac.uk/fsl/fslwiki/PALM). Brain images and statistical maps were visualized using FSLView (version 6.0.5.1, released July 2021, https://fsl.fmrib.ox.ac.uk/fsl/fslwiki/*).* The effects of the primary factor condition on ISCs were assessed in permutation tests for which the GLM consisted of t-tests for pairwise contrasts between conditions. Dependencies between samples (repeated measures) were accounted for by including mean effect regressors for each participant pair and by allowing permutation exchangeability only between samples from the same participant pair across conditions. Whole-brain *z*-maps were FWE-corrected within contrasts and over multiple contrasts. For spatial statistics, threshold-free cluster enhancement (TFCE) was used.

Additional analyses were carried out to assess the predictive power of the proximity of participants’ scores on background measures of their personality, political attitudes and their inter-brain alignment. Multiple regression analyses were carried out, including as predictors the proximity (negative numerical distance) between individual scores on political authoritarianism and political attitudes, and as dependent variable the average ISC of the hemodynamic activity in the conditions *threat* and *blame*, across topics as well as for each topic specifically. For each regression contrast, all other predictors were treated as covariates. Wilks’ lambda permutation tests (1,000 permutations) with tail approximation (*P* < .10) were carried out, using FSL’s PALM (Permutation Analysis of Linear Models; Winkler et al.^74^). Whole-brain *z*-maps were FWE-corrected within contrasts and over multiple contrasts (multiple regressors). For spatial statistics, threshold-free cluster enhancement (TFCE) was used. These regression analyses were carried out after baseline subtraction. This correction aimed at reducing the weight of random baseline correlation between participants, induced e.g., by low-level sensory stimulation external to the experimental stimuli, such as scanner noise. More specifically, for each participant pair, multiple inter-subject correlation maps between unmatched stimulus-specific segments, across frame and topic conditions, were calculated, normalized (Fisher’s *r*-to-*z* transform) and then averaged. Next, for each participant pair, the average baseline correlation map was subsequently subtracted from each normalized stimulus-specific inter-subject correlation map. Finally, all normalized and corrected (baseline subtracted) pairwise correlation maps were then averaged per frame and topic condition and subjected to statistical regression analysis.

## Supplementary Information

Below is the link to the electronic supplementary material.

Supplementary Material 1

## Data Availability

All data, code and materials are available at: https://Github.com/elisavanderplas/PoliticalAttitudes_fMRI.
