# Supplementary material for: Threat and blame frames in political rhetoric about societal issues lead to neural and political polarization
Source: Sci Rep. 2026 Mar 20;16:14304. doi: 10.1038/s41598-026-43389-9 (PMC13144340; doi:10.1038/s41598-026-43389-9)
Supplement: Supplementary file 1 — Supplementary Material 1 [file 41598_2026_43389_MOESM1_ESM.docx]

## **Supplementary Materials**

## **Threat and blame frames in political rhetoric about societal issues lead to neural and political polarization**

Elisa van der Plas^1-2,5*^, Lara Todorova^2^, Karin Heidlmayr^2^, Giedo Jansen^4,5^, Martin Rosema^5^, Alan G Sanfey^2^

^1^Wellcome Centre for Human Neuroimaging, University College London, WC1N 3BG London, United Kingdom
^2^Donders Institute for Brain, Cognition and Behavior, Radboud University Nijmegen, 6525 EN Nijmegen, The Netherlands
^3^Max-Planck Institute for Psycholinguistics, 6525 XD Nijmegen, The Netherlands
^4^Amsterdam Institute for Advanced Labour Studies, Hugo Sinzheimer Instituut, University of Amsterdam, Amsterdam, The Netherlands

^5^Department of Public Administration, University of Twente Enschede, 7522 NB Enschede, The Netherlands
*Corresponding author. Email: elisavanderplas@gmail.com

**These Supplementary Materials include the following:**

Supplementary Materials (5,598 words)

Supplementary Tables S1.1-1.2 and S2.1-5

Supplementary Figures S1 and S2

**STUDY 1
Supplementary Materials 1.1.**

Every video had three versions that each emphasized a different aspect of the societal issue in question, i.e., the personal relevance of its negative outcomes for *threat* frames; it being caused by a third party for blame-frames; and its baseline complexity for neutral frames (e.g., translated in English *“[...] is likely to have a negative impact on your life”, “[...] is caused by poor governance”, or “[...] is a complex problem that is hard to resolve”*, for *threat, blame* and neutral video-clips, respectively; see *Supplementary Table 1.1*). The imagery of the video-clips was kept equal across conditions, only the wording of the video-clips was manipulated at specific points in the video-clips according to the distinct frames. This wording was presented as subtitles in Dutch and with a professional voice-over to account for illiteracy. All sentences obeyed a pre-defined template of ten words in ten sentences, each followed by a jittered inter-sentence interval of 2-4 seconds. The first sentence introduced the main message of the video-clip and was displayed in bold letters in the middle of the screen, after every three sentences the provided information focused on a related, but different consequence of the underlying problem (i.e., increased criminality and loss of culture for immigration, air pollution and flood risks for climate change, and poor elderly care and increased suicide-rates for health care). The final tenth sentence was a take-home message depicting the main idea of the video-clip and an exclamation mark in the *blame* condition; a question mark in the *threat* condition; and full stop for neutral video-clips.

## ***[Supplementary Table 1.1 here]***

**Supplementary Materials 1.2.**

*Political authoritarianism.* We here used a Dutch translation of the traditional authoritarian personality scale (Adorno, 1950) which evaluates a person’s adherence to conventional values; a tendency towards ingroup authority figures, and aggression against people who violate conventional values. Our Dutch authoritarianism scale included three items on political authority (e.g., translated to English “People, and not politicians, need to make most important political decisions” reverse coded) and three items capturing strength (e.g., “The Netherlands needs a strong and courageous leader that people can trust”). Participants rated their agreement with six items on a scale from 1 “completely agree” to 5 “completely disagree” and a separate “I do not know” option from which we computed a main composite score following standard coding procedures (Scheepers et al., 1990).

*Political skepticism scale.* The political skepticism scale measures people’s governmental skepticism (e.g., “Political parties have a good grasp of the problems in our society” reverse coded). A total of six items were rated from 1 “strongly agree” to 5 “strongly disagree” and a separate “I do not know” option from which we computed a main composite score following standard coding procedure (Rutjens et al., 2017).

*Party attitudes.* We rated participant’s attitudes towards the seven main political parties in The Netherlands at the time of recruitment on a 21-point Likert scale from “very unsympathetic” to “very sympathetic” and a separate “I do not know option”. The assessed parties were: CDA, D66, GroenLinks, PvdA, PVV, SP, VVD and 50 Plus. In line with our research question, we only report attitudes towards the Dutch Green Party (GL) and the Dutch Populist Radical Right party (PVV).

*Issue positions.* We tested nine statements that captured the key ideas presented in the video-clips (climate change: “Pollution has negative consequences for our health”; immigration: “Immigrants take the jobs of the Dutch”; health care: “Politicians should invest more money in good health care”) to which participants replied on a scale from 1 “Totally agree” to 5 “Totally disagree” and a separate “I do not know option”. The main composite score captures a participants’ unweighted average agreement with the main idea represented in the video-clip (higher scores represent greater agreement).

*Issue importance.* We recorded how much importance participants assigned to the main views expressed in the video-clips, which were re-coded such that higher ratings indicate greater importance (on a scale from 0 “not important” to 100 “very important”).

## ***[Supplementary Table 1.2 here]***

**Supplementary Materials 1.3.**

We measured negative arousal with each six emotion items, of which three pertained to fearful emotions (“fearful”, “afraid”, and “worried”); and three pertained to anger emotions (“angry”, “irritated” and “hostile”). Responses were given on a PANAS item on a 100-point Likert scale ranging from 0 = “Not at all” to 100 = “Very much”, without any other labels. Following Wagner (2014), we group the six different negative emotions into two groups of three items. This design serves itself for disentangling the effects of fear and anger, without making the assumption that both emotions tap into distinct cognitive processes (Brader & Marcus, 2013: 175). With a factor analysis on the PANAS scores we first extracted a factor comprising positive affect items (happy, inspired, strong, active, enthusiastic, proud) and negative affect items (fearful, angry, worried, afraid, irritated, hostile). We subsequently grouped the negative affect items into two separate factors, pertaining to fear (fearful, worried, afraid) and anger (angry, irritated, hostile). Clustering negative and positive salience was validated by factor analysis (KMO = .79), which validity improved when fear and anger emotions were clustered independently (Crombach’s *a* negative = .73, which is well above the acceptable limit of .30). A post-hoc validation of this cluster supported the separation (Cronbach’s *a*_negative_ = .75, *a*_anger_ = .84 and *a*_fear_ = .75). The clusters of fear and anger emotions used in all analyses was computed as a weighted average of all fear and all anger items.

***[Supplementary Table 1.3 here]***

**Supplementary Materials 1.4.**

Video-clips presented with threat- and blame-frames evoked stronger negative emotion than when the video-clip was presented in its natural neutral (main effect of condition: *F*_2, 1823_ = 8.03, *P* = 0.0003; $\beta_{blamevsneutral}$= -0.27, SE = 0.07, *P* = 0.0001; $\beta_{blamevsthreat}$ = -0.09, SE = 0.06, *P* = 0.14).

Next, we asked whether the distinct frames also evoked distinct negative emotions. The distinct simplification frames did not evoke different levels of fear (main effect of condition on fear: *F*_2,1823_ = 0.98, *P* = 0.38) but did evoke different levels of anger (main effect of condition on anger: *F*_2,1823_ = 18.66, *P* = 9.81e^-09^). Notably, video-clips framed with simplification evoked more anger than when the video-clip was presented neutrally ($\beta_{neutralvs.blame}$= 0.42, SE = 0.07, *P* = 1.29e^-09^; $\beta_{neutralvs.threat}$= 0.22, SE = 0.06, *P* = 2.11e^-04^).

**Supplementary Materials 1.5.**

We first ask whether we could successfully predict participants’ party attitudes (GL, PVV) from the negative emotions that participants experienced while watching video-clips about societal issues. To this end, we entered party attitudes as dependent variables in a linear model with topic condition, negative arousal and their interactions together with the covariates of no interest as predictors. This model shows that participants who experienced more negative arousal while watching immigration video-clips than climate change (β = -0.09, SE = 0.05, *P* = 0.007) or health care (β = -0.12, SE = 0.05, *P* = 0.001) video-clips were more likely to have positive attitudes towards the PVV (interaction effect negative arousal x topic condition: *F*_2,1823_ = 3.61, *P* = 0.027), suggesting that we can infer PVV attitudes from how negatively aroused they feel while they watch evidence about immigration.

We next conduct a similar analysis but with GL attitude as dependent variable—a left-winged party that takes ownership over the issue of climate change. This model revealed that participants who experienced greater negative arousal while watching climate change rather than immigration (β = -0.13, SE = 0.05, *P* = 0.001) or health care video-clips (β = -0.049, SE = 0.05, *P* = 0.366), were more likely to have positive attitudes towards the GL (interaction effect negative arousal x topic condition: *F*_2,1823_ = 3.30, *P* = 0.037). These results again suggest that we can infer people’s political party attitudes from the type of societal issue that evokes strongest negative arousal.

**STUDY 2
Supplementary Materials 2.1.**

Using a linear model to predict trial-by-trial reported negative arousal from threat and blame frames we found that *threat* and *blame* video-clips evoked significantly more negative arousal than the video-clips presented neutrally (main effect of condition: *F*_2, 23_ = 21.44, *P* = 8.08e^-10^). Contrasts showed that this was driven by increased negative arousal following both *threat* ($\beta_{threat}$=10.96, SE = 2.12, *P* = 2.99e^-07^) and blame ($\beta_{blame}$=13.09, SE = 1.99, *P* = 9.87e^-11^) video-clips compared with neutral video-clips. Self-reported fear was not different between video-clips that were framed with *threat* (M = 0.056, SE = 0.20) compared with video-clips that were framed with anger (M = -0.04, SE = 0.20; *t*_24_ = 0.04, *P* = 0.83). Whilst anger was highest for video-clips that were framed with blame (M = 0.056, SE = 0.20) compared with video-clips that were framed with *threat* (M = -0.04, SE = 0.20; *t*_24_ = 127.06, *P* < 2.2e16).

**Supplementary Materials 2.2.**

First, we performed a full factorial analysis with *condition* (*blame, threat*, neutral) and *topic* (immigrant, environment, healthcare) as factors. While we did not find any significant effects for the main effect of the *topic*, or interaction between the *topic* and *frame condition,* we did find a main effect of *frame condition*(see **Supplementary Table 2.1**).

Then we investigated the effect of frame condition with regards to the contrasts of interest: *blame* > neutral, *threat* > neutral, *blame > threat*. While we did not find any significant activation for *blame > threat* condition, we did find significant effects for *threat* > neutral and *blame* > neutral contrasts (see **Supplementary Table 2.2**).

## ***[Supplementary Table 2.1 and 2.2 here]***

**Supplementary Materials 2.3.**

Multiple regression analyses were carried out that aimed at assessing the predictive power of inter-subject proximity in personality or political attitudes on the inter-subject correlations of hemodynamic activity (ISC) in the conditions *threat* and *blame*. Multiple regression analyses aiming at assessing the predictive power of inter-subject proximity in personality or political attitudes on the inter-subject correlations of hemodynamic activity (ISC) in the conditions *threat* and *blame* were also carried out after baseline subtraction (for further detail, see the Methods section in the main manuscript). Coordinates of the maxima in the significant clusters are reported as ‘proximity’ scores, which designate the pairwise negative numerical distance between participants’ scores on background measures of their personality or political attitudes.

Similar to the analyses conducted on right-wing attitudes (PVV) the main manuscript, the proximity between individual scores on left-wing party attitudes was found to be a predictor of the ISC in the *blame* condition and for topic climate change, in the medial frontal cortex (**Supplementary Table 2.3**).

## ***[Supplementary Table 2.3 here]***

After baseline subtraction, the regression analysis additionally revealed that the proximity between individual scores on left-wing GL attitudes was a predictor of medial frontal ISC in the blame condition for topic climate change (**Figs. S2.1**). These findings suggest that the representation of political information with blame causes neural polarization mirroring political attitudes on both the right (PVV) and left (GL) side of the political spectrum. Building upon prior work, this shows that it is not just any negative emotion (Van Baar et al., 2021), but especially blame-related anger that drives political polarization.

## ***[Figs S2.1 here]***

**Supplementary Materials 2.4.**

***Behavioral findings***

In an additional set of analysis, we show that participants with higher scores of political authoritarianism reported to experience less fear in response to the political video-clips (main effect of authoritarianism: *F*_1, 887_ = 5.24, *P* = 0.02, β = -0.19, SE = 0.08, *P* = 2.23e^-02^). Next, we test whether we also find this effect when blame regarding societal issues was manipulated externally by means of the video-clip condition, rather than by internally experienced authoritarianism, as reported in earlier work (Lerner et al., 1998; Vasilopoulos et al., 2019; Vasilopoulou & Wagner, 2017, 2017). We indeed found that video-clips that are presented with blame (which act to shift responsibility to a third party) evoked lower fear than when the video-clips was presented with *threat* (M_blame_ = 37.23, SE = 4.33; M_threat_ = 40.38, SE = 4.63; *t*_24_ = 2.05, *P* = 0.041). This suggests that both internal and external shifts in responsibility can reduce self-reported fear about the issue.

Crucially, and in contrast to the previous finding with fear, participants with higher scores on the trait of political authoritarianism reported more anger in response to the video-clips (main effect of authoritarianism: *F*_1, 887_ = 3.98, *P* = 0.046, β = 0.16, SE = 0.08, *P* = 4.65e^-02^). We observed a similar pattern in response to *threat* framing, finding that anger was higher following *threat* video-clips (M = 43.97, SE = 4.27) than with blame (M = 52.25, SE = 4.18) (dependent samples t-test predicting anger: *t*_24_ = -5.74, *P* = 1.36e^-08^), suggesting that external frames that blame a societal issue on a third party trigger more anger than those frames that only emphasize the *threat* of societal issues. People with higher scores in political authoritarianism generally considered the societal issues to be more important than people with lower scores on this trait (main effect of authoritarianism: *F*_1, 887_ = 45.32, *P* = 3.01e^-11^, β = 0.03, SE = 0.003, *P* = 3.01e^-11^), and less likely to share video-clips (main effect of authoritarianism: *F*_1, 887_ = 12.85, *P* = 0.0004, β = -0.10, SE = 0.03, *P* = 3.57e^-04^).

***Neural findings***

In addition, the proximity between individual scores on the authoritarianism scale was a significant predictor of the ISC in the *threat* condition for topic health care, in the bilateral precentral gyri and in the right juxtapositional lobule cortex (**Fig. S2.2**).

***[Figs. S2.2 here]***

## **Supplementary Figures and Tables**

***Supplementary Table 1.1.*** Original video-clip transcripts per condition.

| **Condition** | **English translation** |
| --- | --- |
| **Video 1a Immigration_neutral_financial** | *1. The Netherlands seems to have no money for immigrants.*  *2. There are 2 million migrants in our country.*  *3. Refugees are often unable to contribute much to our taxes.*  *4. Yet they all need shelter and income.*  *5. It is unclear whether we can continue to pay all migrants.* |
| **Video 1b**  **Immigration_neutral_culture** | *1. Immigration can change our lifestyle*  *2. Immigrants sometimes have different ideas about religion and women's rights.*  *3. How do we ensure that strangers treat each other the way we do?*  *4. It is unclear whether immigration will be at the expense of our culture.*  *5. Immigration may come at the expense of Dutch traditions.* |
| **Video 2a**  **Milieu_neutral_biodiversity** | *1. Climate change is a difficult problem.*  *2. Slowly but surely our nature is becoming more and more gray*  *3. The weather is changing, animals are dying out and there are fewer forests.*  *4. Will this make us increasingly unhappy in The Netherlands?*  *5. We may already be dealing with the negative consequences of climate change.* |
| **Video 2b**  **Milieu_neutral_financial** | *1. Our fuels are becoming increasingly scarce.*  *2. We don't know if our technology can match our consumption.*  *3. It is unclear exactly how extensive the damage is, let alone what we can do about it.*  *4. Raw material consumption could be a threat to The Netherlands.*  *5. To what extent and in what way is still difficult to say.* |
| **Video 3a**  **Health_neutral_financial** | *1. Many are concerned about healthcare in The Netherlands.*  *2. Perfect health care does not exist because that would be unaffordable.*  *3. Therefore, a consideration must be made. As a result, patients increasingly have to pay for their treatment themselves.*  *4. Is it true that the quality of our healthcare is getting worse?*  *5. No one can say for sure.* |
| **Video 3a Health_neutral_independence** | *1. It is difficult to maintain the quality of disease care in The Netherlands.*  *2. Dutch people increasingly pay for the costs themselves.*  *3. This leaves some diseases untreated.*  *4. Family and friends increasingly have to provide part of the care.*  *5. Perhaps cuts in healthcare cannot be prevented.* |
| **Video 1a**  **Immigration_threat_financial** | *1. There is silence about the increasing costs of immigration.*  *2. Migrants all get tax money, houses and jobs.*  *3. What if you can't find a job yourself? Or can't buy a house?*  *4. We will be victims of an immigration policy that we pay for ourselves.*  *5. Immigration will be detrimental to your chances.* |
| **Video 1a**  **Immigration_threat_culture** | *1. Our Netherlands will no longer be The Netherlands in a few years.*  *2. Immigration will make The Netherlands an unsafe Muslim country.*  *3. Strangers will not adopt our social way of life.*  *4. Immigration will therefore be at the expense of your tax money, virtue and openness.*  *5. Our culture will never be the same again.* |
| **Video 2a**  **Milieu_threat_biodiversity** | *1. The Netherlands is becoming increasingly ugly due to climate change.*  *2. Your beautiful environment becomes polluted and withered.*  *3. Your children will never see the parks and animal species that make you so happy now.*  *4.Our nature will be replaced by artificial lawns, apartment buildings and industrial estates.*  *5. The Netherlands will become a dull and gray country due to climate change* |
| **Video 2b**  **Milieu_threat_financial** | *1. High gas consumption will cost The Netherlands dearly.*  *2. It is clear that we do not yet have alternative fuels.*  *3. If fuels run out, industries will grind to a halt and businesses will close.*  *4. No one knows how we will absorb those high costs.*  *5. The next economic crisis will not be long in coming.* |
| **Video 3a**  **Health_threat_independence** | *1. It is just a matter of time before the healthcare system in The Netherlands will collapse.*  *2.Carers are fired and overloaded, patients are neglected.*  *3. If you get sick in The Netherlands, you will be left to your own devices.*  *4. Relationships collapse under the toll that informal care takes on partners and family.*  *5. Will your family take care of you if you become critically ill?* |
| **Video 3b**  **Health_threat_financial** | *1. Dutch healthcare is becoming increasingly unaffordable*  *2. People are afraid of getting sick because healthcare is expensive.*  *3. Cutbacks make hospitals places where you wouldn't want to be caught dead.*  *4.The Dutch are becoming increasingly unhappy*  *5. In your darkest days, you will end up being alone.* |
| **Video 1a Immigration_blame_financial** | *1. In gratitude for our hospitality, immigrants steal from the Dutch.*  *2. We pay tons of tax money every day for integration courses, mosques and illegal refugees.*  *3. We desperately need this money to survive ourselves*  *4. While most foreigners earn dirty money, the Dutch have to make ends meet.*  *5. We would be crazy to let profiteering immigrants use us any longer!* |
| **Video 1b**  **Immigration_blame_culture** | *1. Immigrants are taking away our Dutch culture.*  *2. They invade our country, unceremoniously change our traditions and treat Dutch women without respect.*  *3. Migrants are plotting among themselves against Dutch people and will turn against our customs.*  *4. Immigrants try to manipulate everything we stand for*  *5. We are Sinterklaas! Stay away from our traditions!* |
| **Video 2a**  **Milieu_blame_biodiversity** | *1. Our environmental problems are caused by mass producers.*  *2. Our beaches and parks are being taken away for industrial estates.*  *3. Companies take advantage of our environment, but we are not asked a thing*  *4. Polluting multinationals have the future of The Netherlands in their hands, and then throw it mercilessly to the ground.*  *5. Companies don't give a s* about us! This has to change!* |
| **Video 2b**  **Milieu_blame_financial** | *1. Companies have consumed our fuels.*  *2. Due to their heavy consumption, we will soon be without fuel.*  *3. But companies don't give a s* about you or me, money is all they think about.*  *4.They don't care if we later have to pay a lot more to get to work.*  *5.Selfish companies steal our money! This has to stop now!* |
| **Video 3a**  **Health_blame_culture** | *1. Healthcare institutions are ruining healthcare in The Netherlands.*  *2. They provide fake services and manipulate patients through false information.*  *3. Companies that sell healthcare for profit are only after money.*  *4.They take advantage of the sick to manipulate and abuse them.*  *5.We must do something about institutions that ruin our health!* |
| **Video 3b**  **Health_blame_financial** | *1. Companies are silent about the profit they make at the expense of patients.*  *2. Elderly people are left to their own devices.*  *3. Critically ill patients have to pay for costs they can never afford.*  *4. The sick should be helped, not manipulated for profit.*  *5. Companies are taking advantage of the sick, and this must stop now!* |

***Supplementary Table 1.2.*** Means, standard deviations and ranges for primary measures in Study 1.

| ***Variable*** | ***M (SD)*** | ***Min*** | ***Max*** |
| --- | --- | --- | --- |
| *PANAS positive arousal* | *2.30 (1.71)* | *0.00* | *10.00* |
| *PANAS negative arousal* | *3.72 (2.27)* | *0.00* | *10.00* |
| *PANAS fear* | *7.79 (5.11)* | *1.00* | *21.00* |
| *PANAS anger* | *8.94 (5.13)* | *1.00* | *21.00* |
| *Issue importance: Climate change* | *15.87 (4.41)* | *1.00* | *22.00* |
| *Issue importance: Immigration* | *13.49 (5.30)* | *1.00* | *22.00* |
| *Issue importance: Health care* | *17.99 (3.03)* | *1.00* | *22.00* |
| *Sharing intention* | *2.04 (1.42)* | *1.00* | *6.00* |
| *Party attitudes: PVV* | *5.64 (6.00)* | *1.00* | *22.00* |
| *Party attitudes: GroenLinks* | *11.40 (6.10)* | *1.00* | *22.00* |
| *Big Five – Openness* | *0.88 (1.65)* | *−4.00* | *4.00* |
| *Big Five – Conscientiousness* | *1.29 (1.33)* | *−4.00* | *4.00* |
| *Big Five – Extraversion* | *0.82 (1.50)* | *−4.00* | *4.00* |
| *Big Five – Agreeableness* | *2.41 (1.15)* | *−4.00* | *4.00* |
| *Big Five – Neuroticism* | *−1.00 (1.75)* | *−4.00* | *4.00* |
| *Authoritarianism* | *7.25 (2.55)* | *0.00* | *12.00* |

***Note.*** *M = mean; SD = standard deviation. PANAS subscales reflect summed item scores. Issue importance and party attitudes were measured on 22-point scales unless stated otherwise. Big Five traits are standardized composite scores.*

***Supplementary Table 1.3.* Full Regression Models for Study 1 outcomes (all covariates included)**

| **Predictor** | **PVV attitudes** | **GL attitudes** | **Sharing intention** | **Issue importance** |
| --- | --- | --- | --- | --- |
| Intercept | 5.23*** (1.22) | −0.83*** (0.07) | 0.87*** (0.17) | −0.10*** (0.01) |
| Frame: Blame | −0.16 (0.28) | −0.18 (0.13) | −0.11 (0.17) | −0.12*** (0.02) |
| Frame: Uncertain | 0.12 (0.28) | 0.02 (0.09) | −0.11 (0.17) | −0.00 (0.02) |
| Negative affect | 0.01 (0.06) | 0.06*** (0.01) | 0.01 (0.01) | 0.35*** (0.01) |
| Climate importance | 0.58*** (0.16) | 0.06*** (0.01) | — | — |
| Immigration importance | −0.22*** (0.03) | 0.02** (0.01) | 0.35*** (0.01) | — |
| Health care importance | 0.13* (0.06) | 0.07*** (0.01) | — | — |
| Authoritarianism | −0.53*** (0.06) | 0.07*** (0.01) | — | — |
| Openness | 0.03 (0.10) | 0.10*** (0.02) | 0.03* (0.01) | — |
| Conscientiousness | 0.87*** (0.17) | — | — | — |
| Extraversion | −0.11 (0.17) | — | — | — |
| Agreeableness | −0.18 (0.13) | 0.09** (0.03) | −0.12*** (0.02) | — |
| Neuroticism | 0.02 (0.09) | −0.07*** (0.02) | −0.00 (0.02) | — |

### **Model fit statistics**

| **Statistic** | **PVV** | **GL** | **Sharing** | **Importance** |
| --- | --- | --- | --- | --- |
| *R²* | .41 | .31 | .75 | .75 |
| Adjusted *R²* | .42 | .32 | .75 | .75 |
| *F* | 46.30 | 39.60 | 392.75 | — |
| *p* (model) | < .001 | < .001 | < .001 | < .001 |
| *N* | 1,825 | 1,825 | 1,825 | 1,825 |

### ***Note.*** *Models include all theoretically relevant covariates available for each dependent variable. Unstandardized regression coefficients are shown with standard errors in parentheses. Frame conditions are dummy coded with the reference category defined in the Methods section. All models include demographic and personality covariates as specified. p < .05*, p < .01**, p < .001.*

***Supplementary Table 2.1.*** Means, standard deviations and ranges for primary measures in Study 1.

| **Variable** | ***M* (*SD*)** | **Min** | **Max** |
| --- | --- | --- | --- |
| Appraisal | 2.38 (0.73) | 1.00 | 3.00 |
| PANAS fear | 37.90 (22.41) | 0.00 | 94.33 |
| PANAS anger | 46.47 (22.33) | 0.00 | 98.00 |
| PANAS negative affect | 42.18 (19.04) | 1.17 | 88.00 |
| Issue importance (general) | 65.57 (23.43) | 0.00 | 100.00 |
| Issue importance: Climate change | 84.07 (13.28) | 42.33 | 100.00 |
| Issue importance: Immigration | 31.71 (12.65) | 4.67 | 62.33 |
| Issue importance: Health care | 56.75 (17.29) | 16.33 | 100.00 |
| Sharing intention (video sharing) | 6.01 (7.39) | 0.00 | 39.00 |
| Political authoritarianism | 44.33 (10.40) | 24.83 | 64.67 |
| Political scepticism | 48.82 (11.95) | 23.00 | 71.00 |
| Donation intention | 23.40 (26.43) | 0.00 | 100.00 |

**Note.** *M* = mean; *SD* = standard deviation. Affect measures reflect scaled composite scores. Issue importance, donation intention, and political attitudes were measured on 0–100 scales. Sharing intention reflects observed or self-reported sharing behavior counts.

***Supplementary Table 2.2.*** Regression model results for Study 1

| **Predictor** | **PVV attitudes** | **GL attitudes** | **Video agreement** | **Issue importance** |
| --- | --- | --- | --- | --- |
| Intercept | 5.23*** (1.22) | 11.11*** (1.22) | 20.99*** (4.35) | 67.42*** (13.42) |
| Frame: Blame | −0.16 (0.28) | 1.01* (0.43) | — | — |
| Frame: Uncertain | 0.12 (0.28) | 0.96* (0.43) | — | — |
| Frame: Blame | — | — | −3.25*** (0.78) | −0.45 (2.41) |
| Frame: Threat | — | — | −2.11* (0.82) | −2.00 (2.53) |
| Climate index | −0.23*** (0.06) | 0.58*** (0.16) | — | — |
| Migration index | 0.63*** (0.16) | −0.53*** (0.06) | — | — |
| Health index | −0.01 (0.07) | 0.13* (0.06) | — | — |
| Cynicism index | 0.19*** (0.03) | −0.22*** (0.03) | — | — |
| Authoritarianism index | 0.12* (0.06) | 0.01 (0.06) | — | — |
| Negative affect | 0.34*** (0.08) | 0.03 (0.10) | 0.08*** (0.01) | 0.44*** (0.04) |
| Topic: Environment | 1.41** (0.53) | −0.60 (0.52) | — | — |
| Topic: Health care | 0.97† (0.56) | −0.38 (0.55) | — | — |
| Topic × Negative affect (Environment) | −0.28* (0.13) | 0.23† (0.13) | — | — |
| Topic × Negative affect (Health care) | −0.38** (0.12) | 0.11 (0.12) | — | — |
| Big Five – Agreeableness | 0.07 (0.10) | 0.21* (0.10) | — | — |
| Big Five – Conscientiousness | −0.08 (0.09) | −0.12 (0.09) | — | — |
| Big Five – Extraversion | 0.14† (0.08) | 0.20* (0.08) | — | — |
| Big Five – Neuroticism | −0.14† (0.08) | 0.25*** (0.07) | — | — |
| Big Five – Openness | −0.00 (0.08) | 0.05 (0.08) | — | — |
| Gender | −0.68** (0.25) | 0.08 (0.24) | 0.98† (0.52) | −0.83 (1.59) |
| Age | — | — | −0.33* (0.14) | −1.19** (0.42) |
| Education | −0.35*** (0.10) | 0.02 (0.09) | 0.56 (0.52) | −11.27*** (1.61) |
| Left–right ideology | 0.38*** (0.07) | −0.83*** (0.07) | — | — |
| Political interest | 0.16 (0.17) | 0.87*** (0.17) | — | — |
| Online news reading | −0.04 (0.17) | −0.11 (0.17) | — | — |
| Position: Climate | — | — | −0.04 (0.03) | 0.29*** (0.08) |
| Position: Health care | — | — | −0.01 (0.02) | −0.14** (0.05) |
| Position: Immigration | — | — | 0.02 (0.02) | 0.05 (0.07) |
| Political authoritarianism | — | — | −0.16*** (0.04) | 0.76*** (0.12) |
| Political scepticism | — | — | −0.01 (0.02) | −0.04 (0.07) |

**Model fit statistics**

| **Statistic** | **PVV** | **GL** | **Video agreement** | **Issue importance** |
| --- | --- | --- | --- | --- |
| *R²* | .38 | .42 | .13 | .23 |
| Adjusted *R²* | .37 | .41 | .11 | .22 |
| *F* | 38.41 | 46.30 | 11.48 | 23.39 |
| *p* (model) | < .001 | < .001 | < .001 | < .001 |
| *N* | 1,825 | 1,825 | 897 | 897 |

***Note****. Models include all theoretically relevant covariates available for each dependent variable. Unstandardized regression coefficients are reported with standard errors in parentheses. Frame and topic variables were dummy coded with reference categories specified in the Methods. Interaction terms reflect moderation by negative affect*.

***Supplementary Table 2.3.*** *Significant clusters of BOLD activation for the main effect of condition Results are cluster-corrected using FWE rate of 0.05 and cluster threshold of 10. For each cluster, we present the MNI coordinates (x, y, z) of the local cluster maxima, including the cluster size (k), and cluster label location as indicated in the Harvard-Oxford Cortical Structural Atlas.*

| **k** | **X** | **Y** | **Z** | **Location** |
| --- | --- | --- | --- | --- |
| 1547 | -12 | -96 | 6 | L Occipital Pole |
| 2115 | 20 | -94 | 4 | R Occipital Pole |
| 305 | 6 | -72 | 42 | R Precuneus Cortex |
| 163 | -38 | -86 | -6 | L Lateral Occipital Cortex  (inferior division) |
| 149 | -36 | -50 | -16 | L Temporal Occipital Fusiform Cortex |
| 112 | -46 | -78 | 4 | L Lateral Occipital Cortex (inferior division) |
| 88 | 38 | -56 | -18 | R Temporal Occipital Fusiform Cortex |
| 83 | -44 | -52 | 56 | L Angular Gyrus |
| 51 | 44 | -58 | 50 | R Lateral Occipital Cortex (superior division) |
| 10 | 24 | -40 | -14 | R Temporal Occipital Fusiform Cortex |
| 16 | 4 | 34 | 28 | Paracingulate Gyrus |
| 31 | 0 | -22 | 30 | Cingulate Gyrus (posterior division) |

***Supplementary Table 2.4.*** *Significant clusters of BOLD activation for the contrasts of interest. Results are cluster-corrected using FWE rate of 0.05 and cluster threshold of 10. For each cluster, we present the MNI coordinates (x, y, z) of the local cluster maxima, including the cluster size (k), and cluster label location as indicated in the Harvard-Oxford Cortical Structural Atlas.*

| **Contrast** | **K** | **X** | **Y** | **Z** | **Location** |
| --- | --- | --- | --- | --- | --- |
| Threat > Neutral | 323 | 6 | -74 | 42 | R Precuneus |
|  | 105 | -46 | -50 | 54 | L Supramarginal Gyrus  (posterior division) |
|  | 33 | 50 | -54 | 52 | R Angular Gyrus |
|  | 10 | -52 | -36 | 50 | L Supramarginal Gyrus  (anterior division) |
|  | 10 | -42 | -22 | 52 | L Postcentral Gyrus |
| Blame > Neutral | 139 | 8 | -72 | 42 | R Precuneus |
|  | 11 | -42 | -48 | 48 | L Supramarginal Gyrus  (posterior division) |
|  | 18 | -6 | -68 | 42 | L Precuneus |
|  | 18 | 2 | 34 | 28 | Paracingulate Gyrus |
|  | 11 | 46 | -58 | 52 | R Lateral Occipital Cortex |
|  | 17 | 2 | -24 | 28 | Cingulate Gyrus |

***Supplementary Table 2.5. Significant clusters of ISC multiple regression analyses after baseline subtraction.*** *For each cluster, we present the MNI coordinates (x, y, z) of the cluster maxima, including the cluster size (k), and cluster label location as indicated in the Harvard-Oxford Cortical Structural Atlas, the Harvard-Oxford Subcortical Structural Atlas, and a Probabilistic cerebellar atlas (MNI). For spatial statistics, threshold-free cluster enhancement (TFCE) was used. Whole-brain z-maps were FWE-corrected within contrasts and over multiple contrasts (multiple regressors). Information on cluster size and coordinates is given for a significance threshold P<0.05 except when indicated with an asterisk (*), where a threshold of P<0.10 was used for visualization. A cluster size threshold of 10 was applied. T – threat, B – blame; HE - Health care, CL – Climate change, IM – Immigration.*

| **Contrast** | **k** | **x** | **y** | **z** | **Location** |
| --- | --- | --- | --- | --- | --- |
| **Left-wing party attitude / B_CL*** | 111 | 6 | 54 | 22 | R Frontal Pole |
| **Political authoritarianism / T_HE** | 197 | 4 | -14 | 66 | R Juxtapositional Lobule Cortex (formerly Supplementary Motor Cortex); Precentral Gyrus |
|  | 44 | -16 | -16 | 74 | L Precentral Gyrus |


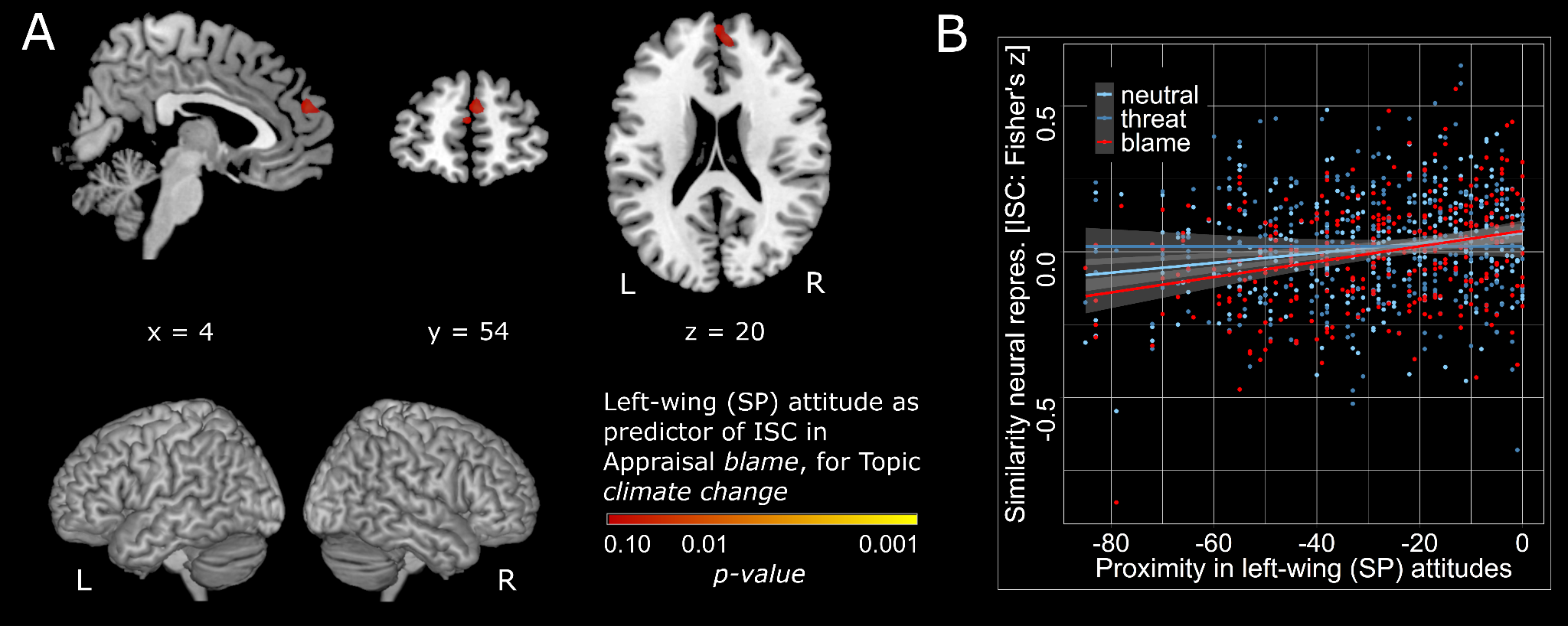


***Figs S2.1. Regression between background measures and inter-subject correlations (ISC): Green Party (GL) party attitude / blame, for Topic climate change. A.*** *The proximity (negative numerical distance) between individual scores on left-wing party attitude as a predictor of the ISC in the blame* *condition and for topic climate change is depicted. Whole-brain significance (P-value) map, FWE-corrected within and across contrasts; after baseline subtraction. Despite reaching the significance threshold, data are shown for P < .10 for visibility.* ***B.*** *The proximity (negative numerical distance) between individual scores on left-wing party attitudes (SP) as a predictor of the ISC in the neutral/blame/threat* *condition and for topic climate change is depicted. Values are extracted for the significance mask obtained for blame (see panel A).*


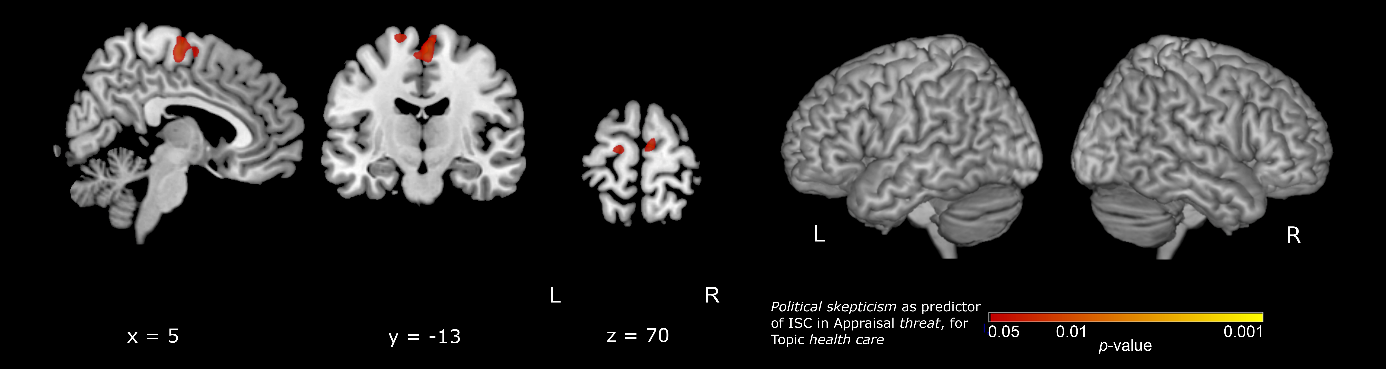


***Figs S2.2. Regression between background measures and inter-subject correlations (ISC): Political authoritarianism / threat,*** ***for Topics health care.*** *The proximity (negative numerical distance) between individual scores on political authoritarianism as a predictor of the ISC in the threat* *condition and for topic health care is depicted. Whole-brain significance (P-value) map, FWE-corrected within and across contrasts.*
